# Räumlicher Zugang zu Palliativstationen in Deutschland: Integrierte Analyse von Verfügbarkeit und Erreichbarkeit mittels E2SFCA-Methode

**DOI:** 10.1007/s00103-025-04124-3

**Published:** 2025-09-03

**Authors:** Theresa Petzold, Friedemann Nauck, Christian Banse, Jobst Augustin, Maximiliane Jansky

**Affiliations:** 1 Klinik für Palliativmedizin, Universitätsmedizin Göttingen (UMG), Georg-August-Universität Göttingen, Von-Siebold-Straße 3, 37075 Göttingen, Deutschland; 2 Institut für Versorgungsforschung in der Dermatologie und bei Pflegeberufen (IVDP), Universitätsklinikum Hamburg-Eppendorf (UKE), Hamburg, Deutschland

**Keywords:** Stationäre Palliativversorgung, Zugang zu Gesundheitsleistungen, Deutschland, Geografische Informationssysteme, Floating Catchment Area-Methode, Palliative care units, Health services accessibility, Germany, Geographic information systems, Floating catchment area method

## Abstract

**Einleitung:**

Demografischer Wandel und steigende Patient*innenzahlen sind Herausforderungen für einen wohnortnahen Zugang zu Palliativstationen. Verfügbarkeit und Erreichbarkeit als Dimensionen des räumlichen Zugangs, in bisherigen Studien meist isoliert voneinander betrachtet, werden mittels der Methode „Enhanced Two-Step Floating Catchment Area“ (E2SFCA) integriert, um durch ein realistischeres Bild der Versorgungssituation regionale Unterschiede zu identifizieren.

**Methoden:**

Auf Gemeindeebene wurde mithilfe der E2SFCA-Methode ein Zugangsindex (*Z*_*i*_) berechnet. Grundlage bildeten Daten zur Bevölkerung, zu Bettenkapazitäten und zur Erreichbarkeit innerhalb von 30 min Fahrzeit. Der Index wurde in Quintile unterteilt und den Kategorien niedriger (Q1, Q2), mittlerer (Q3) und hoher Zugang (Q4, Q5) zugeordnet.

**Ergebnisse:**

Der Zugang zu den 372 in Deutschland identifizierten Palliativstationen variiert stark zwischen und innerhalb von städtischen und ländlichen Gebieten. In dünn besiedelten Gebieten führen Versorgungsangebote zu einem überdurchschnittlichen Zugang. Zugleich entstehen in weiter entfernten Gemeinden lange Fahrzeiten (Beispiel Mecklenburg-Vorpommern). Städtische Regionen erreichen durch eine hohe Angebotsdichte häufig hohe Zugangsindices. Eine hohe Bevölkerungsdichte führt bei bestehendem Angebot zu einem durchschnittlichen Zugang (Beispiel Nordrhein-Westfalen).

**Diskussion und Fazit:**

Die meisten Menschen in Deutschland könnten Palliativstationen in 30 min erreichen, mit regional großen Unterschieden. Die erstmals für Palliativstationen angewandte E2SFCA-Methode bietet eine präzisere Analyse als Studien, die sich an landkreisbezogenen Bettenkapazitäten und Einwohnerzahlen orientieren. Um die tatsächliche Versorgung abzubilden, muss die Versorgungssituation in Gebieten mit schlechtem Zugang detailliert untersucht werden.

## Abstract (en)

**Introduction:**

Demographic change and rising patient numbers pose challenges for local access to palliative care units. Availability and accessibility as dimensions of spatial access, which have mostly been considered separately in previous studies, are integrated using the method “Enhanced Two-Step Floating Catchment Area” (E2SFCA) in order to identify regional differences by providing a more realistic picture of the care situation.

**Methods:**

At the community level, an access index (*Z*_*i*_) was calculated using the E2SFCA method. It was based on population data , bed capacity, and accessibility within a 30-minute drive. The index was divided into quintiles and assigned to categories with low (Q1, Q2), medium (Q3), and high access index (Q4, Q5).

**Results:**

Access to the 372 palliative care units identified in Germany varies greatly between and within urban and rural areas. In sparsely populated areas, care services lead to above-average access. At the same time, long travel times arise in more remote communities (e.g., Mecklenburg-Western Pomerania). Urban regions often achieve high access indices due to a high density of services. A high population density results in average access when services are available (e.g., North Rhine-Westphalia).

**Discussion and conclusion:**

Most people in Germany could reach palliative care units within 30 min, with large regional differences. The E2SFCA method, applied for the first time to palliative care units, offers a more precise analysis than studies based on district-specific bed capacities and number of inhabitants. In order to reflect the actual regional care situation, the care provision in areas with poor access must be examined in detail.

## Hintergrund

Eine würdevolle Begleitung in der letzten Lebensphase sollte für jeden Menschen unabhängig von Wohnort, finanziellen Mitteln oder Herkunft möglich sein [1]. Vor dem Hintergrund des demografischen Wandels könnte die Umsetzung des Anspruchs jedoch zu einer Herausforderung werden. Mit einem medianen Alter von 47,8 Jahren hat Deutschland die viertälteste Bevölkerung weltweit [2]. Im Zeitraum zwischen 2009 bis 2050 wird die Anzahl der Sterbefälle um 25 % steigen. Gleichzeitig versterben Patient*innen aufgrund des anhaltenden Fortschritts medizinischer Behandlungsmöglichkeiten in einem zunehmend hohen Lebensalter [3]. Sowohl die Inzidenz an Tumorerkrankungen als auch die Anzahl von nichtonkologischen Patient*innen mit unheilbaren Erkrankungen steigt. Daraus kann abgeleitet werden, dass auch der Bedarf an palliativmedizinischer Versorgung ansteigen wird [4].

In Deutschland existieren zahlreiche ambulante und stationäre, allgemeine und spezialisierte palliative Versorgungsstrukturen. Die allgemeine Palliativversorgung (APV) leistet eine Basisversorgung durch Hausärzt*innen, Pflegedienste, Pflegeheime und Krankenhäuser unter Einbeziehung ambulanter Hospizdienste bei niedriger Komplexität. Bei hoher Komplexität der Patient*innensituation erfolgt die Behandlung durch spezialisierte ambulante Teams (SAPV) in Ergänzung zu den oben genannten Strukturen der APV oder im Rahmen der stationären spezialisierten Palliativversorgung durch Palliativstationen, Palliativdienste und stationäre Hospize. Palliativstationen behandeln Patient*innen in Krisensituationen, bei komplexer Symptombelastung oder häuslicher Dekompensation für wenige Tage bis mehrere Wochen multiprofessionell. Die Linderung belastender Symptome, die Stabilisierung des Zustandes, die Verbesserung der Lebensqualität sowie die Organisation einer ambulanten Weiterversorgung sollen durch multiprofessionelle Teamarbeit erreicht werden, in die Ärzt*innen, Pflegefachpersonen, Physiotherapeut*innen und psychosoziale Berufsgruppen einbezogen sind. Für die Weiterversorgung sind tragfähige ambulante und stationäre Angebote wie SAPV und Hospize notwendig, sodass die Versorgungsstrukturen ambulant und stationär idealerweise eng verzahnt arbeiten [5–7].

Das seit 2015 geltende Hospiz- und Palliativgesetz soll den flächendeckenden Ausbau palliativer und hospizlicher Versorgungsstrukturen fördern und den Anspruch von Bürger*innen auf einen gerechten Zugang zur wohnortnahen Palliativversorgung sichern [8]. Der Zugang zu Gesundheitsleistungen wird als mehrdimensionales Konzept verstanden. Neben der Unterscheidung in potenziellen und realisierten Zugang [9] werden die Dimensionen der Verfügbarkeit (Availability), Erreichbarkeit (Accessibility), Kompatibilität (Accomodation), Erschwinglichkeit (Affordability) und Akzeptanz (Acceptability) am häufigsten in diesem Kontext genannt [10]. Die Dimensionen *Verfügbarkeit* und *Erreichbarkeit* beschreiben räumliche Aspekte des potenziellen Zugangs und können zum Begriff des „räumlichen Zugangs“ zusammengefasst werden.

Der räumliche Zugang gilt als wichtiger Indikator für die Qualität von Gesundheitsleistungen und die Bedarfsdeckung [9–12]. Die Erreichbarkeit im Kontext der Palliativstationen schließt sowohl den Hin- und Rückweg vom Wohnort der Patient*innen, in der Regel mit Krankentransport- oder Rettungsfahrzeugen, als auch die Besuchswege ihrer Angehörigen ein. Lange Anfahrtswege können von Patient*innen und An- und Zugehörigen als belastend wahrgenommen werden [13–15] und Besuchsmöglichkeiten einschränken [16]. Gleichzeitig kann eine lange Fahrzeit zum Heimatort aufgrund der hohen Belastung eine (heimatnahe) Verlegung erschweren.

Für die palliativmedizinische Versorgung existieren in Deutschland keine Vorgaben zur maximalen Fahrzeit. Einzig die Qualitätsanforderungen für onkologische Zentren bieten einen gesetzlich verankerten Orientierungswert. Sie werden durch den Gemeinsamen Bundesausschuss (G-BA) zur Konkretisierung der besonderen Aufgaben von Zentren und Schwerpunkten gemäß § 136c Absatz 5 SGB V (Zentrums-Regelungen) festgeschrieben. Hier wird als eine strukturelle Anforderung eine „24-stündige Verfügbarkeit palliativmedizinischer Versorgung am Standort des onkologischen Zentrums innerhalb von 30 min am Bett der Patientin oder des Patienten“ genannt [17]. Weitere Orientierung bieten die in Deutschland und im europäischen Ausland verwendeten Werte aus wissenschaftlichen Studien [18–20]. Da in palliativen Versorgungssituationen häufig eine instabile Patient*innensituation vorliegt, kann zudem bei Fahrzeiten ab 30 min von einem zunehmenden Leidensdruck ausgegangen werden [19, 21].

Die Verfügbarkeit von Palliativstationen wird üblicherweise als Verhältnis der vorhandenen Betten zur Bevölkerungszahl innerhalb administrativer Einheiten (z. B. Landkreise) berechnet. Als Orientierung dienen die Empfehlungen der European Association for Palliative Care (EAPC) und der Deutschen Gesellschaft für Palliativmedizin (DGP), die auf Palliativstationen 50 Betten je 1 Mio. Einwohner*innen vorsehen [22]. Nicht berücksichtigt wird dabei jedoch die Erreichbarkeit der Angebote.

Im Gutachten zur Weiterentwicklung der ambulanten Bedarfsplanung von 2018 wurden Gravitationsmodelle – auch bekannt als Floating Catchment Area-Methoden (FCA-Methoden) bzw. erreichbarkeitsgewichtete Verfügbarkeit – als am besten geeignete Analysemethode herausgearbeitet [23]. Die Limitationen der häufig eindimensionalen Erreichbarkeitsanalysen lassen sich überwinden, wenn neben der Erreichbarkeit auch die Verfügbarkeit berücksichtigt und flexible Einzugsgebiete anstelle von festgelegten administrativen Einheiten genutzt werden. Der Aspekt der Verfügbarkeit wird über die Anzahl der Palliativstationen, inkl. ihrer potenziellen Bettenkapazitäten, abgebildet und in Relation zur Bevölkerung als potenzielle Patientengruppe gesetzt. Dies erfolgt nicht anhand festgelegter administrativer Einheiten, sondern innerhalb flexibler Einzugsgebiete, die durch eine definierte Fahrzeit bestimmt sind und den Aspekt der Erreichbarkeit abbilden. Befinden sich potenzielle Patient*innen in überlappenden Einzugsgebieten mehrerer Standorte, erfolgt die Zuordnung nicht ausschließlich zum nächstgelegenen Versorgungsangebot. Ein Abgleich mit den von der EAPC empfohlenen Verhältniszahlen entfällt dadurch.

Auch wenn die Anzahl der verfügbaren Angebote in den vergangenen Jahren stetig angestiegen ist, ist unklar, ob ein flächendeckender Zugang zu Palliativstationen erreicht ist. In Deutschland existieren bereits einige Studien [24–28], welche die Erreichbarkeit oder Verfügbarkeit palliativmedizinischer Versorgungsangebote untersucht haben. Diese beziehen sich vorwiegend auf einzelne Bundesländer und ausgewählte Versorgungsangebote. Gesell et al. analysierten 2023 erstmals die spezialisierte Palliativversorgung deutschlandweit unter Berücksichtigung der Fahrzeit und der Bevölkerungsverteilung, wobei potenzielle Bettenkapazitäten der Palliativstationen nicht mit abgebildet wurden [18]. Allen Studien ist die isolierte Betrachtung von Verfügbarkeit und Erreichbarkeit als Dimensionen des räumlichen Zugangs gemeinsam.

Ziel der hier vorliegenden Studie ist daher, den räumlichen Zugang zu Palliativstationen mithilfe der Methode „Enhanced Two-Step Floating Catchment Area“ (E2SFCA) zu analysieren und damit ein realistischeres Bild für dieses Versorgungssetting zu zeichnen. Die E2SFCA-Methode ist eine Weiterentwicklung der klassischen 2SFCA-Methode (Two-Step Floating Catchment Area) zur Erfassung der räumlichen Erreichbarkeit von Gesundheitsdiensten. Sie fügt dem ursprünglichen Modell eine Gewichtung nach Entfernungen hinzu. Bei der Analyse sollen Gebiete, in denen Palliativstationen nicht innerhalb von 30 min Fahrzeit erreichbar sind, sowie mögliche Unterschiede in der Versorgung zwischen ländlichen und städtischen Regionen identifiziert werden.

## Methoden

### Datenbasis und Datenvorbereitung

#### Palliativstationen und Bettenkapazitäten.

Die Identifizierung der Palliativstationen, inklusive deren Bettenkapazitäten, erfolgte zunächst auf Basis des Wegweisers Hospiz- und Palliativversorgung (WHPV; [29]), einer frei zugänglichen webbasierten Selbstauskunftsdatenbank der DGP. Diese beinhaltet zahlreiche stationäre und ambulante palliativmedizinische und hospizliche Versorgungseinrichtungen. Weitere Einrichtungen und deren Bettenkapazitäten wurden durch die Listen der Landesverbände des Deutschen Hospiz- und Palliativverbandes (DHPV) identifiziert. Alle Angaben zu identifizierten Einrichtungen wurden manuell überprüft (Stand April 2022).

#### Bevölkerungsdaten.

Für die kartografische Darstellung wurde auf die Flächengeometrien aus den Verwaltungsgebietsdaten (VG250-Geometrien) des Bundesamtes für Kartografie und Geodäsie (BKG) aus dem Jahr 2019 zurückgegriffen, die mit Bevölkerungsdaten auf Gemeindeebene verknüpft wurden [30]. Für die E2SFCA-Methode ist eine Umwandlung der flächenhaft (polygon) vorliegenden Bevölkerungsdaten in einen Punkt notwendig, der repräsentativ für die Fläche (Gemeinde) sein sollte. Der VG250-Datensatz enthält mit den „Gemeindekernen“ den Bevölkerungsschwerpunkt, der, im Unterschied zu dem klassischerweise verwendeten geometrischen Schwerpunkt der Fläche, den Ort abbildet, an dem die meisten Menschen der Gemeinde leben.

### Datenanalyse

Zur Untersuchung des räumlichen Zugangs wurde die E2SFCA-Methode angewandt [31]. Die Erreichbarkeit fließt als Fahrzeit mit dem Pkw von maximal 30 min ein [17–21]. Die Berechnung eines Zugangsindex erfolgte in einem 2‑stufigen Verfahren, in dem zunächst ein Angebots-Nachfrage-Verhältnis *PPR*_*j*_  für jede Palliativstation und anschließend ein Zugangsindex *Z*_*i*_ für jeden Bevölkerungsschwerpunkt kalkuliert wurde.

#### Schritt 1.

**Abb. 1:**
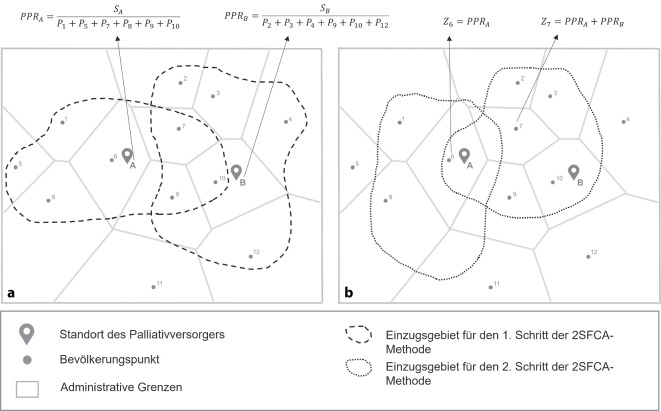
Schematische Darstellung der E2SFCA-Methode unter Verzicht auf die Unterteilung des Einzugsgebietes in Subzonen, inkl. deren Gewichtungen (= 2SFCA-Methode), mit Bildung der Angebots-Nachfrage-Verhältnisse *PPR*_*A,B*_ in Schritt 1 (**a**) und Berechnung der Zugangsindices *Z*_6,7_  in Schritt 2 (**b**). *2SFCA* Two-Step Floating Catchment Area, *E2SFCA* Enhanced Two-Step Floating Catchment Area

Für die Berechnung des Angebots-Nachfrage-Verhältnisses *PPR*_*j*_ wurde für jede Palliativstation *j* ein maximales Einzugsgebiet (*d*_*max*_), hier Pkw-Fahrzeit 30 min, festgelegt und mithilfe des Network Analyst, einem Werkzeug zur Analyse von Straßen- und Versorgungsnetzen, kalkuliert. Dieser maximale Radius wurde in 3 Subzonen (*r*_*1,2,3*_ = 0–10 min, 10–20 min, 20–30 min) unterteilt. Folgend wurden alle Bevölkerungsschwerpunkte *i* innerhalb der Subzonen ($d_{ij}$≤ *d*_*max*_) identifiziert und aufsummiert (∑*P*_*i*_). Die Summe wurde danach ins Verhältnis mit den Bettenkapazitäten der Palliativstationen *S*_*j*_ gesetzt und ein gewichtetes Angebots-Nachfrage-Verhältnis *PPR*_*j*_ gebildet (für jede Subzone wird ein Gewicht *W*_*r*_ festgelegt). Die Vorgehensweise ist in Abb. 1a vereinfacht dargestellt, unter Verzicht auf die Darstellung der Subzonen und Gewichtungen (= 2SFCA-Methode).

$$PPR_{j}=\frac{S_{j}}{\begin{array}{c}\sum \limits_{i\in \left\{d_{ij}\in r=1\right\}}P_{i}W_{1}\\+\sum \limits_{i\in \left\{d_{ij}\in r=2\right\}}P_{i}W_{2}\\+\sum \limits_{i\in \left\{d_{ij}\in r=3\right\}}P_{i}W_{3}\end{array}}$$

Die Gewichtung wird durch eine Entfernungsabnahmefunktion abgebildet, welche auf einer Gauß’schen Normalverteilung beruht. Weiter von den Palliativstationen entfernte Bereiche fließen mit einem geringeren Gewicht ein und bilden so den Zugang realistischer ab. Die Wahl der Gewichtung orientiert sich an der Originalpublikation von Luo und Qi [31]. Für die Analysen im Rahmen der vorliegenden Arbeit wurde eine starke Entfernungsabnahmefunktion gewählt (1,0; 0,42 und 0,03), welche die Autor*innen für Analysen von Angeboten der Basisversorgung (Hausärzt*innen, Apotheken) empfehlen. Obwohl Palliativstationen eine spezialisierte Versorgungsform darstellen, wird die Zumutbarkeit weiter Fahrzeiten für die Patient*innengruppe und ihre Angehörigen als eher gering eingestuft.

#### Schritt 2.

Im zweiten Arbeitsschritt wurde ein Zugangsindex (*Z*_*i*_) gebildet. Dabei wurde für jeden Bevölkerungsschwerpunkt *i* ein Fahrzeitradius von 30 min, ebenfalls gegliedert in 3 Subzonen, kalkuliert. In der Folge wurden alle Betten-Einwohner-Verhältnisse *PPR*_*j*_ unter Verwendung der Gewichtung innerhalb der 3 Subzonen aufsummiert. Abschließend wurde der *Z*_*i*_ für jeden Bevölkerungsschwerpunkt *i *auf Gemeindeebene gebildet (Abb. 1b). Der *Z*_*i*_ ist umso höher, je mehr Betten es auf Palliativstationen gibt, je schneller die Stationen erreichbar sind und je geringer die Bevölkerungsanzahl im 30 min Radius ist.

$$\begin{aligned}Z_{i}&=\sum \limits_{j\in \left\{d_{ij}\in r\equiv 1\right\}}PPR_{j}W_{1}\\&+\sum \limits_{j\in \left\{d_{ij}\in r\equiv 2\right\}}PPR_{j}W_{2}*\\&+\sum \limits_{j\in \left\{d_{ij}\in r\equiv 3\right\}}PPR_{j}W_{3}\\\end{aligned}$$

Die Ergebnisse wurden als Quintile dargestellt (Q1–Q5). Gemeinden, die über keinen Zugang innerhalb von 30 min zu Palliativstationen verfügen, bilden eine eigene zusätzliche Kategorie („kein Zugang“). Die Quintile wurden, orientiert an der Literatur [32, 33] in einen niedrigen (Q1 und Q2), mittleren (Q3) und hohen Zugangsindex (Q4 und Q5) unterteilt.

### Kategorisierung anhand des Gemeindetyps

Die Gemeinden wurden nach Bevölkerungsgröße und zentralörtlicher Funktion gemäß der Stadt- und Gemeindetypisierung des Bundesinstituts für Bau‑, Stadt- und Raumforschung (BBSR) kategorisiert (Groß‑, Mittel-, größere Kleinstädte, kleine Kleinstädte und Landgemeinden; [34]).

Alle Analysen wurden mit dem GIS-Programm ArcGIS Pro 2.9 (Esri Inc., Kalifornien, Redlands, USA; GIS = geografisches Informationssystem) durchgeführt.

## Ergebnisse

**Abb. 2:**
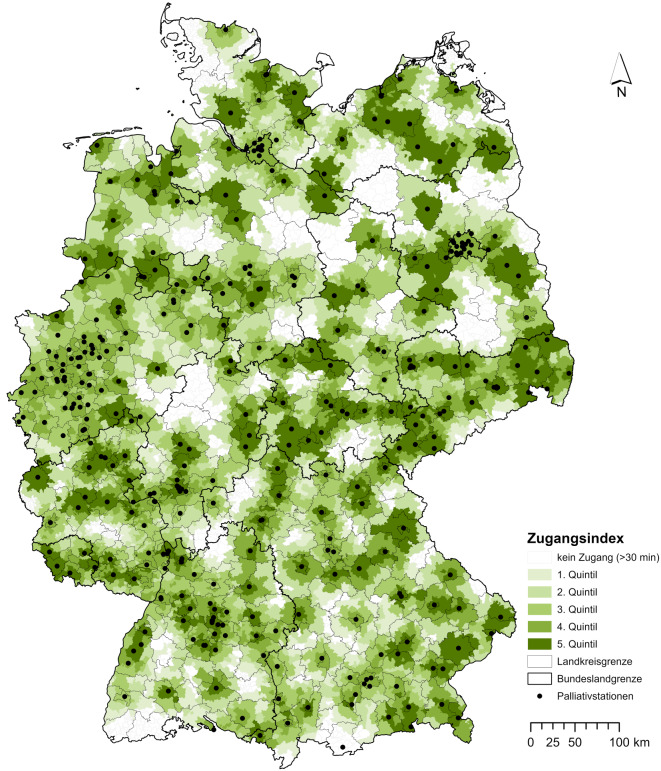
Deutschlandweiter Zugangsindex (*Z*_*i*_) auf Gemeindeebene für Palliativstationen, unterteilt in die Quintile: niedriger (Q1, Q2), mittlerer (Q3) und hoher Zugang (Q4, Q5). *Datenquelle*: Flächengeometrien der Verwaltungsgebiete und Bevölkerungsdaten: VG250-Geometrien des Bundesamtes für Kartografie und Geodäsie (BKG), Standorte der Palliativstationen: Wegweiser Hospiz- und Palliativversorgung (WHPV) und Listen der Landesverbände des Deutschen Hospiz- und Palliativverbandes (DHPV)

Insgesamt wurden im Recherchezeitraum April 2022 *N* = 372 Palliativstationen mit *N* = 3230 Betten und einer durchschnittlichen Bettenkapazität von 9 (Spanne 2–32) im Rahmen der spezialisierten stationären Palliativversorgung identifiziert. Die meisten Versorgungseinrichtungen (*n* = 338) waren im WHPV verzeichnet, *n* = 34 wurden nach weiteren Recherchen hinzugefügt. In Abb. 2 ist die Lokalisation der Palliativstationen und der *Z*_*i*_ auf Gemeindeebene für Deutschland visualisiert.

**Abb. 3:**
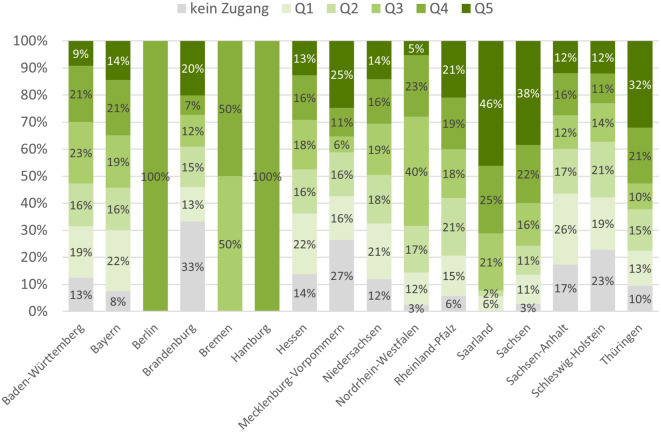
Anteil der Gemeinden mit niedrigem (Q1, Q2), mittlerem (Q3) und hohem Zugang (Q4, Q5) zu Palliativstationen nach Zugangsindex (*Z*_*i*_) innerhalb der einzelnen Bundesländer. *Datenquelle*: Flächengeometrien der Verwaltungsgebiete und Bevölkerungsdaten: VG250-Geometrien des Bundesamtes für Kartografie und Geodäsie (BKG), Standorte der Palliativstationen: Wegweiser Hospiz- und Palliativversorgung (WHPV) und Listen der Landesverbände des Deutschen Hospiz- und Palliativverbandes (DHPV)

Insgesamt zeigt sich ein heterogenes Muster. In Agglomerationsräumen wie dem Ruhrgebiet oder den Großräumen Stuttgart, Berlin oder Hamburg ist eine hohe Konzentration von Palliativstationen zu verzeichnen. Abb. 3 zeigt die Ergebnisse nach Anteil der Gemeinden in den Zugangsquintilen, aufgegliedert nach den Bundesländern.

Das Saarland und Sachsen weisen einen hohen Anteil von Gemeinden mit hohem Zugangsindex auf (71 % bzw. 60 % in Q4 und Q5), keine bzw. nur 3 % der Gemeinden haben keinen Zugang innerhalb von 30 min. Auch in Thüringen haben 53 % der Gemeinden einen hohen Zugangsindex (*Z*_*i*_ in Q4 und Q5), während sich die übrigen Gemeinden annährend gleich auf die weiteren Kategorien verteilen. Große Unterschiede innerhalb des jeweiligen Bundeslandes gibt es in Brandenburg und Mecklenburg-Vorpommern. Während viele Gemeinden in Brandenburg (33 %) und Mecklenburg-Vorpommern (27 %) keinen Zugang zu einer Palliativstation innerhalb von 30 min haben, hat ein weiteres Drittel (Mecklenburg-Vorpommern: 36 %) bzw. Viertel (Brandenburg: 27 %) einen hohen *Z*_*i*_. Bundesländer mit einem hohen Anteil von Gemeinden mit niedrigem *Z*_*i*_ oder ohne Zugang in 30 min sind Sachsen-Anhalt (43 % bzw. 17 %) und Schleswig-Holstein (40 % bzw. 23 %). Ein Beispiel für Bundesländer mit einem großen Anteil an Gemeinden mit einem mittleren *Z*_*i*_ sind Nordrhein-Westfalen (40 % in Q3) sowie der Stadtstaat Bremen (50 % in Q3, 50 % in Q4). In den übrigen Bundesländern (Baden-Württemberg, Bayern, Hessen und Niedersachsen) ist die prozentuale Verteilung der Gemeinden nach *Z*_*i*_-Quintil in etwa gleichmäßig.

**Abb. 4:**
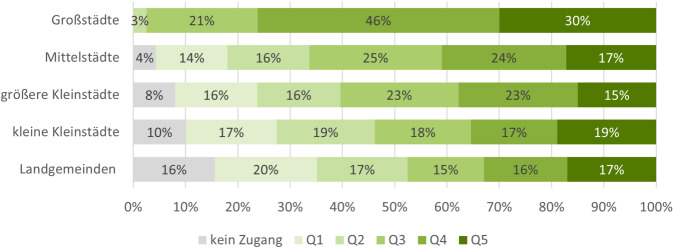
Anteil der Gemeinden mit niedrigem (Q1, Q2), mittlerem (Q3) und hohem Zugang (Q4, Q5) zu Palliativstationen nach Zugangsindex (*Z*_*i*_) und Stadt- und Gemeindetyp des Bundesinstituts für Bau‑, Stadt- und Raumforschung (BBSR). *Datenquelle*: Flächengeometrien der Verwaltungsgebiete und Bevölkerungsdaten: VG250-Geometrien des Bundesamtes für Kartografie und Geodäsie (BKG), Standorte der Palliativstationen: Wegweiser Hospiz- und Palliativversorgung (WHPV) und Listen der Landesverbände des Deutschen Hospiz- und Palliativverbandes (DHPV)

Großstädte verfügen über einen höheren *Z*_*i*_ als andere Gemeindetypen (Abb. 4): 76 % der großstädtischen Gemeinden weisen einen *Z*_*i*_ im obersten und zweitobersten Quintil auf. Großstädtische Gemeinden mit einem niedrigen *Z*_*i*_ gibt es kaum. Bei den Gemeinden der Mittelstädte und größeren Kleinstädte machen mittlere Werte des *Z*_*i*_ (jeweils 25 % und 23 % in Q3) und höhere Werte (jeweils 24 % und 23 % in Q4) die größten Anteile aus. In der Kategorie der kleinen Kleinstädte und Landgemeinden ist die prozentuale Verteilung der Gemeinden nach Quintil in Bezug auf den *Z*_*i*_  annähernd gleichmäßig. Es wird jedoch deutlich, dass der Anteil an Gemeinden, die über keinen Zugang zu einer Palliativstation innerhalb von 30 min verfügen, mit zunehmender Ländlichkeit ansteigt. Bei großstädtischen Gemeinden liegt dieser Anteil bei 0 %, in den weiteren Kategorien nimmt er stetig zu bis 16 % bei den Landgemeinden.

## Diskussion

Die vorliegende Arbeit zeigt bundesweite Ergebnisse zum räumlichen Zugang zu Palliativstationen unter Berücksichtigung der Dimensionen Verfügbarkeit und Erreichbarkeit. Insgesamt verfügt Deutschland über einen sehr guten Zugang zu Palliativstationen. In einem Großteil der Gemeinden kann eine Mehrheit der Bevölkerung innerhalb von 30 min Fahrzeit mit dem Pkw eine Palliativstation erreichen.

In dieser Arbeit wird erstmals die E2SFCA-Methode im Bereich der palliativen Versorgungsangebote angewandt und damit ein gravitationsbasierter, komplexerer Ansatz zur Untersuchung des räumlichen Zugangs zu Versorgungseinrichtungen genutzt. Dies stellt eine Weiterentwicklung gegenüber früheren Studien dar, die sich wie auch nationale und internationale Standards – etwa jene der EAPC [6] – primär auf Verfügbarkeitskennzahlen wie Betten-Einwohner-Verhältnisse stützen [25, 26, 35–37]. Erreichbarkeit wird dabei in Form von einfachen Entfernungen als Luftlinien betrachtet [38]. Eine der ersten deutschlandweiten Analysen unter Einbeziehung geografischer Methoden in Form von Netzwerkanalysen lieferten Gesell et al. (2023). Hier konnte, wie in der vorliegenden Studie, eine gute Erreichbarkeit von Palliativstationen beschrieben werden: Mehr als drei Viertel der Bevölkerung in Deutschland erreichen eine stationäre Einrichtung innerhalb von 30 min mit dem Pkw [18].

Gravitationsbasierte Verfahren wurden bisher nur auf Ebene einzelner Bundesländer angewandt – etwa in Sachsen, wo seit 2013 der „Hospiz- und Palliativbericht Sachsen“ veröffentlicht wird, zuletzt im Jahr 2022. Anstelle der Gesamtbevölkerung wird hier auf die Anzahl der Verstorbenen zur Berechnung eines Zugangsindex zurückgegriffen [24]. Der dort beschriebene „hohe Zugang“ zu stationärer Palliativversorgung kann auch in der vorliegenden Studie bestätigt werden, da 76 % der Gemeinden über einen mittleren bis hohen Zugangsindex und nur 3 % über keinen Zugang innerhalb von 30 min Fahrzeit verfügen.

In der vorliegenden Studie wurden die Daten zu Palliativstationen aus dem WHPV, die auch früheren Analysen auf nationaler [35] und auf Bundeslandebene (z. B. Rheinland-Pfalz [36] oder Mecklenburg-Vorpommern [26]) zugrunde lagen, durch weitere Recherche ergänzt und zusätzliche Angebote identifiziert. Daher ist davon auszugehen, dass frühere Studien die tatsächliche Versorgung unterschätzten.

Da sich der räumliche Zugang innerhalb Deutschlands stark unterscheidet, ist es sinnvoll, sowohl regionale als auch bundesweite kleinräumige Analysen durchzuführen. Während regionale Studien, wie für Sachsen vorliegend, Besonderheiten von lokalen Versorgungsnetzwerken berücksichtigen können, ermöglichen deutschlandweite Studien die Beachtung von Mitversorgungsbeziehungen über Bundeslandgrenzen hinweg. Dies ist insbesondere für Patient*innen und deren Angehörige in Bundeslandgrenzgebieten relevant, die Versorgungseinrichtungen unabhängig von administrativen Zugehörigkeiten aufsuchen [23, 33].

Durch die Zuordnung der Gemeinden zum Stadt- und Gemeindetyp des BBSR (Bundesinstitut für Bau‑, Stadt- und Raumforschung) konnten Unterschiede im räumlichen Zugang zwischen Stadt und Land verdeutlicht werden. Großstädte verfügen über einen guten Zugang, da sich hier Angebote stationärer Palliativversorgung konzentrieren. Mit zunehmender Ländlichkeit steigt der Anteil an Gemeinden, die über keinen Zugang zu einer Palliativstation innerhalb von 30 min verfügen. In den in großen Teilen ländlich geprägten Bundesländern Brandenburg und Mecklenburg-Vorpommern kristallisieren sich deshalb große Unterschiede beim räumlichen Zugang heraus. Während einige kleinere Gemeinden überdurchschnittlich gut versorgt sind, bestehen in anderen erhebliche Defizite, was den Zugang zu Palliativstationen betrifft. Dies lässt sich durch die Berücksichtigung des potenziellen Bedarfs anhand der Bevölkerungsdichte in Gravitationsmodellen erklären. Wenn es in den betreffenden Bundesländern viele ländlich geprägte Gemeinden und kleine Kleinstädte mit einer eher geringen Bevölkerungsanzahl gibt, in denen eine Palliativstation lokalisiert ist, stellt sich dies rasch als ein überdurchschnittlicher Zugang für die Bevölkerung dar. Gleichzeitig existieren in einigen Gemeinden dieser Kategorie keine Palliativstationen, weshalb die dort wohnhaften Menschen lange Fahrzeiten zur nächsten Palliativstation auf sich nehmen müssen, was zu einem niedrigen *Z*_*i*_ führt. In Nordrhein-Westfalen, das durch eine hohe Dichte an Agglomerationsräumen geprägt ist, werden entgegen der Erwartung eher mittlere Zugangswerte erreicht. Dies lässt sich durch die große Bevölkerungsdichte mit vielen potenzielle Patient*innen erklären, die auf die vorhandenen Versorgungseinrichtungen trifft. Ein Stadt-Land-Gefälle, das in einigen anderen Studien formuliert wurde [18, 27, 36], kann also nur teilweise bestätigt werden und muss je nach Anzahl der Versorgungsangebote und Bevölkerungsdichte differenziert betrachtet werden.

### Implikationen für die Versorgungspraxis

Eine würdevolle, professionelle Behandlung und Begleitung in einer lebensverkürzenden Erkrankungssituation sollte für alle Menschen zugänglich sein. Da die Aufnahme auf Palliativstationen häufig in Krisensituationen mit akuter Symptombelastung erfolgt, können lange Transportwege eine hohe Belastung für Patient*innen darstellen. Sie wirken zudem direkt auf die Versorgungsqualität: Da die Patient*innen für den Transport auf Mittel des Rettungsdienstes angewiesen sind – Krankentransportwagen (KTW) oder Rettungstransportwagen (RTW) –, werden Ressourcen gebunden, die in der Akutversorgung fehlen. Gleichzeitig gibt es auch im Bereich der präklinischen Notfallversorgung große Diskussionen hinsichtlich der Gewährleistung einer flächendeckenden Versorgung aufgrund steigender Einsatzzahlen bei zunehmendem Personalmangel [39, 40]. Ziel der Behandlung auf einer Palliativstation ist in der Regel die Rückverlegung in die Häuslichkeit oder eine heimatnahe stationäre Versorgungseinrichtung (Pflegeheim, Hospiz). Lange Transportwege stellen hier einen hohen Anspruch an die Transportfähigkeit und die Stabilität der Patient*innen und können eine Verlegung in die Häuslichkeit und Weiterversorgung gefährden.

Ebenso bedeutend für die Stabilisierung schwerkranker Patient*innen ist die Einbeziehung des unmittelbaren sozialen Umfelds. Kurze Fahrzeiten sind für An- und Zugehörige wichtig, da sie die Patient*innen in einer instabilen Krankheits- oder in der letzten Lebensphase besuchen und emotional, psychisch und existenziell unterstützen möchten. Darüber hinaus werden Angehörige häufig als Vorsorgebevollmächtigte in den Entscheidungsprozess zu Therapiezieländerungen mit einbezogen und sind für eine erfolgreiche Symptomlinderung mitentscheidend [41]. Eine besonders große Belastung stellen lange Fahrzeiten für Angehörige dar, die weitere Verpflichtungen wie minderjährige Kinder im Haushalt oder die Pflege eines weiteren Familienangehörigen haben.

Abzuwarten bleibt, welche Auswirkungen aktuelle gesundheitspolitische Entwicklungen im Rahmen der Krankenhausstrukturreform auf Palliativstationen haben werden. Eine Zentralisierung und Spezialisierung der Klinikstruktur könnte eine wohnortnahe Palliativversorgung gefährden. Eine Schließung kleinerer Kliniken mit Palliativstationen könnte den räumlichen Zugang in ländlichen Regionen weiter erschweren und das Versorgungsgefälle zwischen Stadt und Land verschärfen [42, 43]. Die Versorgungssituation ist komplex und muss über die stationäre palliativmedizinische Behandlung und Begleitung hinaus betrachtet werden. Eine abgestimmte Palliativversorgung, die ambulante und stationäre sowie allgemeine und spezialisierte Strukturen integriert, kann die Betreuung der Patient*innen verbessern. Aufgrund der besonderen Versorgungsziele in der ambulanten und stationären (spezialisierten) Palliativversorgung bleibt jedoch unklar, in welchem Umfang diese Strukturen als Kompensationsmechanismen fungieren können. Auch könnte der insgesamt gestiegene Druck auf das Gesundheitssystem vor allem im Bereich der APV eher zu einem größeren Bedarf an spezialisierter (stationärer) Palliativversorgung führen [44, 45].

### Stärken und Limitationen

Hervorzuheben ist, dass erstmals eine Methode aus dem FCA-Spektrum in der Palliativversorgung in Deutschland angewandt wurde. Hierbei wurden beide Dimensionen des räumlichen Zugangs – Verfügbarkeit und Erreichbarkeit – unter Einbezug der Bettenkapazitäten von Palliativstationen sowie der Bevölkerungsdaten berücksichtigt. Bei den Bettenkapazitäten handelt es sich jedoch um die *potenzielle* Kapazität einer Station. Die tatsächliche Belegungssituation, mögliche Kapazitätseinschränkungen durch z. B. Personalmangel oder Wartezeiten von Patient*innen aufgrund hoher Auslastung können nicht abgebildet werden. Die vorliegende Arbeit zeigt zudem, dass die Eintragungen im WHPV, auf denen viele Arbeiten zur Hospiz- und Palliativversorgung aufbauen, lückenhaft sein können.

Die Verwendung von kleinräumigeren Bevölkerungsdaten, z. B. auf Gitterzellebene (1 km- oder 100 m-Raster) kann insbesondere in dicht besiedelten Gebieten zu präziseren Ergebnissen führen. Zum Zeitpunkt der Analyse lagen die Zensusdaten 2022 auf Gitterzellebene noch nicht vor. Diese hätten auch den Einbezug von weiteren soziodemografischen Merkmalen, wie den Anteil der über 65-Jährigen in der Bevölkerung, und damit eine präzisere Abbildung potenzieller Palliativpatient*innen ermöglicht. Da solche Daten zum Analysezeitpunkt nicht auf Gemeindeebene verfügbar waren, wurde auf die Gesamtbevölkerung zurückgegriffen, was angesichts regionaler Unterschiede in der Altersstruktur zu Verzerrungen führen kann.

Limitierend ist außerdem, dass die Analysen ausschließlich für den Pkw als Transportmittel durchgeführt wurden. Gerade die ältere Bevölkerung ist in ihrer Mobilität mit dem Pkw oft eingeschränkt. Um differenzierte Aussagen treffen zu können, ist die Einbeziehung des öffentlichen Personennahverkehrs (ÖPNV) notwendig, was besonders in ländlichen Gebieten zu erheblichen Unterschieden in den Ergebnissen führen kann. Zudem spiegeln die berechneten Fahrzeiten nur die theoretische Erreichbarkeit wider. Reale Hindernisse wie Ampeln, Staus oder Witterung fließen dabei nicht ein. Auch wird angenommen, dass Patient*innen ein wohnortnahes Angebot innerhalb von 30 min Fahrzeit nutzen, was in der Realität nicht immer der Fall ist. Die Integration von Patient*innen in Versorgungsnetzwerke, z. B. onkologische Patient*innen an Universitätskliniken, kann zu einer Vergrößerung der Einzugsgebiete führen. Nicht zuletzt kann auch die Einordnung von 30 min als maximale Fahrzeit diskutiert werden. Eine Sensitivitätsanalyse mit einer Erweiterung der maximalen Fahrzeit könnte hier weitere Informationen zum räumlichen Zugang liefern. Zudem könnte eine Untersuchung unter Einbeziehung der o. g. weiteren Dimensionen des Zugangs zu Gesundheitsleistungen den Rahmen dieser Studie ergänzen.

## Fazit

Die vorliegende Untersuchung zeigt für viele Regionen in Deutschland einen guten Zugang zu Palliativstationen. Durch die Einbeziehung des Stadt- und Gemeindetyps des BBSR konnten Unterschiede im Zugang zwischen Stadt und Land verdeutlicht werden. Die verwendete Methodik kann dabei helfen, Versorgungslücken zu entdecken und diese unter Einbeziehung von weiteren regionalen palliativen Versorgungsangeboten näher zu untersuchen. Um eine bedarfsgerechte wohnortnahe Versorgung sicherzustellen, sollten sich künftige Planungsansätze nicht allein an Bettenkapazitäten orientieren. Neben Faktoren der Erreichbarkeit und alternativen Versorgungsangeboten sollten demografische Besonderheiten (z. B. regional hohes Durchschnittsalter) oder sozioökonomische Faktoren (z. B. Anteil an Single-Haushalten) stärker in den Fokus rücken [46]. Auch Patient*innenpräferenzen hinsichtlich der Versorgungssettings sollten beachtet werden. Quantitative und qualitative Befragungen von Betroffenen zu tatsächlich zurückgelegten Fahrzeiten und deren Zumutbarkeit könnten zusätzliche wertvolle Informationen liefern. Zudem sollten Daten zur Inanspruchnahme und zum Bedarf einbezogen werden, um Regionen mit Über- oder Unterversorgung zu identifizieren und schließlich zu einer Verbesserung der Planung von Angeboten der stationären Palliativversorgung beizutragen.

## Data Availability

Die während der vorliegenden Studie erzeugten und/oder analysierten Datensätze sind auf begründete Anfrage unter folgender Adresse erhältlich: palliativ.forschung@med.uni-goettingen.de.
